# Chondrocytes Proliferation of Patients with Cartilage Lesions in Their Own Body for Use in Cartilage Tissue Engineering: Hypotheses on a New Approach for the Proliferation of Autologous Chondrocytes

**DOI:** 10.31661/gmj.v8i0.1483

**Published:** 2019-08-14

**Authors:** Zahra Abpeikar, Mostafa Soleimannejad, Akram Alizadeh

**Affiliations:** ^1^Department of Tissue Engineering, School of Advanced Technologies, Shahrekord University of Medical Sciences, Shahrekord, Iran; ^2^Department of Tissue Engineering and Applied Cell Sciences, Faculty of Medicine, Semnan University of Medical Sciences, Semnan, Iran

**Keywords:** Chondrocyte, Cell Proliferation, Cartilage, Tissue Regeneration, Osteoarthritis

## Abstract

Osteoarthritis is one of the most common chronic diseases, which have involved 250 million people around the world. One of the challenges in the field of cartilage tissue engineering is to provide an adequate source of chondrocytes to prevent changes in gene expression profile as a result of multiple passages.We hypothesized that by creating a low invasive lesion by scalpel or shear laser in the outer ear cartilage and stimulation of wound healing process, hyperplasia occurs and will provide an appropriate number of autologous chondrocytes for extraction and use in articular cartilage tissue engineering. Also, due to the effect of platelet-rich plasma and biomechanical forces in stimulating and accelerating of the repair process, these two factors can be used to achieve more desirable results.We describe a new approach to proliferate chondrocytes in the body. To evaluate this idea, various techniques of gene expression at the level of RNA or protein and animal experiments for histological studies can be used. Also, flowcytometry technique can be used to determine the cell viability and counting them.The use of autologous cell sources with minimal changes in gene expression profile can be promising in tissue engineering products.

## Introduction

Osteoarthritis (OA) is the most common type of arthritis that affects millions of people around the world. This condition occurs when the protective cartilage at the end of the bone disappears over time. Despite the simple structure of cartilage tissue, it has little ability for repair and regeneration for reasons such as lack of vessels, highly differentiated cells, the low population density of cells, and slow replacement of matrix. Medical and rehabilitation therapies such as physiotherapy only are useful in the early and middle stages of OA, but in advanced cases, only surgery and autologous transplantation can be performed. More than one million different types of cartilage surgery are performed annually around the world, and half of them are often led to the replacement of damaged cartilage [1-3]. Today, tissue engineering strategies have brought new hopes for cartilage repair. A lot of researches have been done on the use of chondrocytes in articular cartilage repair. As a result of problems associated with this cells such as lack of access, limited number, de-differentiation, and loss of morphology [4], researchers have focused on other cellular sources, such as mesenchymal stem cells (MSCs) [5]. However, many studies have shown that chondrocytes have a higher potential than MSCs for the formation of hyaline cartilage. Therefore, in many tissue engineered constructs, chondrocytes are used. To obtain a sufficient number of chondrocytes, 200 to 300 mg of biopsy is needed.Also, isolated cells should be cultured and passaged at least four times–although this depends on the initial density [6, 7]. During culture, chondrocytes lose their phenotype; in other words, they undergo de-differentiation and get fibroblastic morphology. Also, their size and collagen matrix secretion reduce. Microarray analysis identified changes in the expression of 137 genes in chondrocytes after two passage[8]. These changes are mainly downregulation of superficial zone protein, bone morphogenetic proteins-2 (BMP-2), transforming growth factor-β1 (TGF-β1), fibroblast growth factor receptor-3, cartilage oligomeric matrix protein, aggrecan, collagen II, collagen XI, collagen IX,*SOX 9*, and upregulation of mesenchymal or fibroblastic genes including collagen I, collagen X, collagen III, tenascin, and versican[2]. In a study, it was found that these changes occur within four days [9]. Thus with each passage, the gene expression profile of cells change, and these changes affect the formation of neocartilage. Therefore, a method to expand chondrocytes without such changes would be very helpful.The purpose of this article was to present an idea or hypothesis for the proliferation of chondrocytes in the patient’s body. The two main factors in the growth and repair of articular cartilage include growth factors and mechanical forces [10]. Studies have shown that the TGF-β family, especiallyBMPs, cartilage-derived morphogenetic proteins, osteogenic proteins, and growth differentiation factors, have a significant effect on the growth and repair of bone and cartilage tissues [11]. In addition to growth factors, it has been determined that mechanical forces stimulate the synthesis of extracellular matrix proteins in vitro and in vivo, thus can affect the overall structure of the tissue. In the absence of mechanical forces, tissues like cartilage are atrophied, so researchers add mechanical stimuli to tissue engineering processes[10]. In general, tissue engineering processes of articular cartilage in ex vivo are different from other tissues, because chondrocytes require mechanical stimuli for differentiation. The presence of mechanical forces such as hydrostatic pressure or direct compression stimulates cells to secrete more extracellular matrix (ECM) compared to static culture. Many studies have been done to understand what forces are useful for the culture of chondrocytes, and according to their results, bioreactors and certain processes have been designed to utilize the advantage of mechanical stimuli. The most common bioreactors use hydrostatic pressure and direct or shear pressure (caused by fluid) to stimulate chondrocytes[12]. Application of cyclic tensile force with low frequency and low intensity on chondrocytes increases the synthesis of proteoglycans in vitro while increasing the intensity and frequency of applied force has reverse effects and acts to degrade and reduce matrix synthesis [13].Also, the results showed that cyclic tensile force on chondrocyte cells up to 3% strain, 0.17 HZ and 2 hours, had no biological effects or had very poor responses in vitro, while application of cyclic tensile force between 3–10% strains, 0.17–0.5 Hz, and 2–12 hours showed anabolic effects on ECM synthesis. But cyclic tensile force above 10% strain, 0.5 Hz, and 12 hours apply destructive and catabolic effects on the ECM [14]. The effects of growth factors in repair of injuries and lesions have led to the hypothesis that platelet-rich plasma (PRP), which has these factors, can stimulate the repair process in cartilage. Studies have shown that microvesicles and exosomes in platelets contain many growth factors. The most important growth factors in the platelets are a platelet-derived growth factor, TGF-β, fibroblast growth factor, insulin-like growth factor-1, connective tissue growth factor, epidermal growth factor, and hepatocyte growth factor. These factors are widely used for the treatment of OA [15].Also, there are various micro RNAs in the platelets; it is said that some of them, like microRNA-23b,play a role in the differentiation of MSCs into chondrocytes[16].Studies have shown that ligament healing has been increased by intra-articular injection of miRNA 210 in small animal models[17]. Also, platelet concentrates have anti-inflammatory properties that are critical for tissue repair, specially cartilage tissue[18]. Therefore, due to the effective role of growth factors and mechanical stimuli in the process of cartilage repair, PRP (as a source of growth factors) and mechanical forces such as tensile stress can be used to stimulate the repair process in the cartilage tissue.

## Presentation of the Hypothesis

Many researchers are looking for solutions to reduce changes in the gene expression profile and to maintain cell phenotype of chondrocytes in multiple cell passages. These approaches in tissue engineering can have positive effects on the transfer of products from the laboratory to the clinic. We are looking for an approach to the proliferation of chondrocytes in the body to minimize changes in the gene expression profile and to maintain the chondrocyte phenotype. This approach can have beneficial results in the formation of neocartilage. For this purpose, we assumed that by creating a low invasive lesion in the outer ear cartilage, following the process of normal repair of the lesion, hyperplasia and an increase in the number of chondrocytes occur in the external ear cartilageso an appropriate number of autologous chondrocytes for use in cartilage tissue engineering will be provided. Therefore, the purpose of this hypothesis is to provide an acceptable number of autologous chondrocytes without in vitro passages for use in cartilage tissue engineering. Studies have shown that the formation of sharp cartilage lesions prevents widespread cell death in comparison with blunt lesions in the animal model. In this way, by reducing cell death, matrix secretion will be more effective to facilitate the process of integration with the surrounding tissue. Therefore, better repair occurs in sharp lesions [19, 20]. Also, by strong shear laser, very delicate slices can be created. Powerful lasers deliver very high energy to the tissue, which ultimately converts to heat energy in the tissue; this heat can cause tissue damage and subsequently tearing or cutting of tissue. This laser feature is used in surgery to enhance the accuracy of surgery, because laser light can cut out the tissue like a delicate blade of surgery, and it helps the surgeon to do proper surgery in place where it is difficult to move surgeon’s hands or in areas where very thin thickness of slices are required such as eye surgeries, skin peeling, vascular lesions, laparoscopy, etc. Bleeding in laser surgery is very low due to the thermal coagulability of the laser,and the repair speed of the wound is also high. This speed of wound healing is not related to the laser’s thermal effect but depends on cellular optical stimulation, which can cause vital activity of the cell and stimulate the repair process. This laser effect is called biological stimulation caused by cold or low-energy lasers. In other words, to achieve this stimulating effect, it is not necessary to have a powerful laser [21, 22]. Therefore, to create a sharp lesion, according to the results of previous studies, a shear laser or a scalpel can be used to create sharp lesion.

Also, due to the important role of PRP (as a source of growth factors) and biomechanical forces (such as tensile stress with low frequency and low intensity) in repair process, these factors can be used to stimulate repair process and to achieve a more desirable outcome.

## Testing the Hypothesis

To examine this hypothesis by performing animal studies on dog or rabbit, nine experimental groups can be considered as follows:

1. Control

2. Creation of lesion by shear laser

3. Creation of lesion by scalpel

4. Creation of lesion by shear laser with PRP injection in situ

5. Creation of lesion by scalpel with PRP injection in situ

6. Creation of lesion by shear laser along with low intensity and low frequency of tensile stress at the site of the lesion (between 3–10% strains, 0.17–0.5 Hz, and 2–12 hours)

7. Creation of lesion by scalpel along with low intensity and low frequency of tensile stress at the site of the lesion

8. Creation of lesion by shear laser along with low-intensity and low frequency of tensile stress and PRP injection together at the site of the lesion

9. Creation of lesion by scalpel along with low-intensity and low frequency of tensile stress and PRP injection together at the site of the lesion

**Figure 1:**
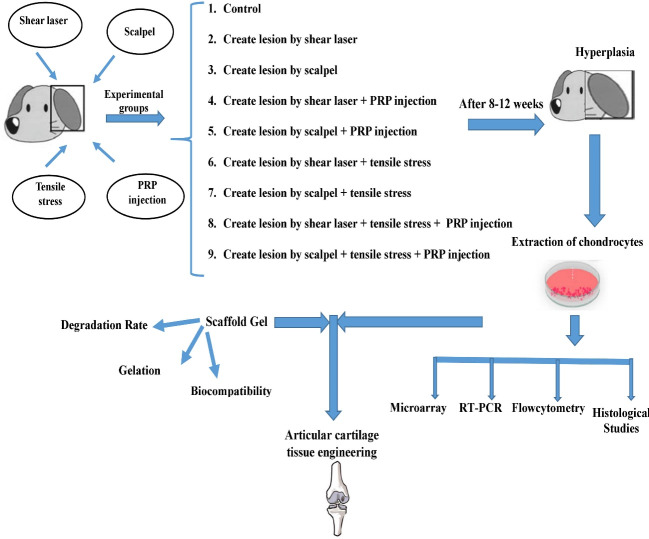
Presentation and testing hypothesis

The effect of these factors on the process of repair and cartilage hyperplasia at different periods (4, 8, and 12 weeks after the lesion) and in different groups can be investigated by sampling from the wound site, extracting and counting chondrocytes (Figure-1). The following techniques can be used for further investigation and comparison of results in different groups with each other and with the control group:

1. Characterization, counting, and evaluation of cell viability by flow cytometry

2. Microarray analysis to determine the number and type of modified genes.

3. Examining of chondrocyte genes expression at the level of RNA or protein.

4. Histological studies

Also, by microarray analysis and examination of chondrocyte genes expression in cells derived from multiple passages, which is commonly used for the proliferation of chondrocytes in cartilage tissue engineering constructs, the results can be compared with the results of animal studies in the hypothesis.

## Implications of the Hypothesis

This hypothesis was presented to introduce a suitable method for the proliferation of chondrocytes in the body as a live bioreactor and to overcome challenges in providing of adequate and appropriate cell sources for cartilage tissue engineering and to minimize changes in the gene expression profile of chondrocytes. We hope that this hypothesis will have positive outcomes in the transferring of cartilage tissue engineering products to the clinic.

## Conclusion

We expect to achieve a sufficient source of chondrocytes by creating a low invasive lesion in outer ear cartilage and stimulation of chondrocytes proliferation in the body. In this way, we will minimize the changes in the gene expression profile that occur in conventional methods of cell proliferation as a result of multiple passages.

## Conflict of intrest

The authours have no conflict of interest.

## References

[1] Beigi MH, Atefi A, Ghanaei HR, Labbaf S, Ejeian F, Nasr-Esfahani MH (2018). Activated platelet-rich plasma improves cartilage regeneration using adipose stem cells encapsulated in a 3D alginate scaffold. J Tissue EngRegen Med.

[2] Huang BJ, Hu JC, Athanasiou KA (2016). Cell-based tissue engineering strategies used in the clinical repair of articular cartilage. Biomaterials.

[3] Toh WS, Lai RC, Hui JHP, Lim SK, editors. MSC exosome as a cell-free MSC therapy for cartilage regeneration: implications for osteoarthritis treatment. Semin Cell Dev Biol; 2017: Elsevier. 10.1016/j.semcdb.2016.11.008 27871993

[4] Melero-Martin JM, Al-Rubeai M (2007) In Vitro Expansion of Chondrocytes. In: Ashammakhi N, Reis R, Chiellini E, editors. Topics in Tissue Engineering 2007;chapter 1,pp. 1–37 .

[5] Mobasheri A, Kalamegam G, Musumeci G, Batt ME (2014). Chondrocyte and mesenchymal stem cell-based therapies for cartilage repair in osteoarthritis and related orthopaedic conditions. Maturitas.

[6] Barbero A, Grogan S, Schäfer D, Heberer M, Mainil-Varlet P, Martin I (2004). Age related changes in human articular chondrocyte yield, proliferation and post-expansion chondrogenic capacity. Osteoarthritis Cartilage.

[7] Stenhamre H, Slynarski K, Petren C, Tallheden T, Lindahl A (2008). Topographic variation in redifferentiation capacity of chondrocytes in the adult human knee joint. Osteoarthritis Cartilage.

[8] Ma B, Leijten JCH, Wu L, Kip M, van Blitterswijk C, Post JN (2013). Gene expression profiling of dedifferentiated human articular chondrocytes in monolayer culture. Osteoarthritis Cartilage.

[9] Barlic A, Drobnic M, Malicev E, Kregar-Velikonja N (2008). Quantitative analysis of gene expression in human articular chondrocytes assigned for autologous implantation. J Orthop Res.

[10] Darling EM, Athanasiou KA (2003). Biomechanical strategies for articular cartilage regeneration. Ann Biomed Eng.

[11] Erlacher L, Ng CK, Ullrich R, Krieger S, Luyten FP (1998). Presence of cartilage-derived morphogenetic proteins in articular cartilage and enhancement of matrix replacement in vitro. Arthritis Rheum.

[12] Darling EM, Athanasiou KA (2003). Articular cartilage bioreactors and bioprocesses. Tissue Eng.

[13] Fukuda K, Asada S, Kumano F, Saitoh M, Otani K, Tanaka S (1997). Cyclic tensile stretch on bovine articular chondrocytes inhibits protein kinase C activity. J Lab Clin Med.

[14] Bleuel J, Zaucke F, Brüggemann G-P, Niehoff A (2015). Effects of cyclic tensile strain on chondrocyte metabolism: a systematic review. PLoS One.

[15] Marmotti A, Rossi R, Castoldi F, Roveda E, Michielon G, Peretti GM. PRP and articular cartilage: a clinical update. Biomed Res Int 2015;2015. 10.1155/2015/542502 PMC4436454 26075244

[16] Ham O, Song B-W, Lee S-Y, Choi E, Cha M-J, Lee CY (2012). The role of microRNA-23b in the differentiation of MSC into chondrocyte by targeting protein kinase A signaling. Biomaterials.

[17] Shoji T, Nakasa T, Yamasaki K, Kodama A, Miyaki S, Niimoto T (2012). The effect of intra-articular injection of microRNA-210 on ligament healing in a rat model. Am J Sports Med.

[18] Bendinelli P, Matteucci E, Dogliotti G, Corsi MM, Banfi G, Maroni P (2010). Molecular basis of anti-inflammatory action of platelet-rich plasma on human chondrocytes: Mechanisms of NF-κB inhibition via HGF. J Cell Physiol.

[19] Redman S, Dowthwaite G, Thomson B, Archer C (2004). The cellular responses of articular cartilage to sharp and blunt trauma. Osteoarthritis Cartilage.

[20] Nishiwaki H, Fujita M, Yamauchi M, Isogai N, Tabata Y, Kusuhara H (2017). A Novel Method to Induce Cartilage Regeneration with Cubic Microcartilage. Cells Tissues Organs.

[21] Mattioli F, Presutti L, Caversaccio M, Bonali M, Anschuetz L (2017). Novel Dissection Station for Endolaryngeal Microsurgery and Laser Surgery: Development and Dissection Course Experience. Otolaryngol Head Neck Surg.

[22] Farivar S, Malekshahabi T, Shiari R (2014). Biological effects of low level laser therapy. J Lasers Med Sci.

